# Prognostic factors in locally advanced oesophageal squamous cell carcinoma: a clinical and radiomic analysis of neoadjuvant immunochemotherapy before surgery

**DOI:** 10.3389/fonc.2025.1508477

**Published:** 2025-03-13

**Authors:** Yan Zhu, Zhenzhong Zhang, Shuangqing Chen, Genji Bai, Qingqing Xu, Lili Zhang, Max Gao, Aichao Ruan, Lili Guo

**Affiliations:** ^1^ Department of Radiology, the Affiliated Huai’an No.1 People’s Hospital of Nanjing Medical University, Huai’an, Jiangsu, China; ^2^ Department of Thoracic Surgery, the Affiliated Huai’an No.1 People’s Hospital of Nanjing Medical University, Huai’an, Jiangsu, China; ^3^ Department of Radiology, the Affiliated Suzhou Hospital of Nanjing Medical University, Suzhou Municipal Hospital, Gusu School, Nanjing Medical University, Suzhou, Jiangsu, China; ^4^ Department of Pathology, the Affiliated Huai’an No.1 People’s Hospital of Nanjing Medical University, Huai’an, Jiangsu, China; ^5^ Computer Science and Engineering, University of California, Davis, Davis, United States

**Keywords:** locally advanced oesophageal squamous cell carcinoma, neoadjuvant immunochemotherapy, radiomics scores, cox regression, survival prediction

## Abstract

**Background:**

The treatment of locally advanced oesophageal squamous cell carcinoma (LAESCC) without distant metastasis remains a subject of debate. Neoadjuvant immunochemotherapy (NIC) combined with surgery is the preferred initial approach for managing LAESCC. However, information on the clinical efficacy and survival of patients with LAESCC treated with NIC followed by surgery is limited.

**Methods:**

This retrospective analysis aimed to identify predictors NIC treatment effectiveness and on patient survival. We developed a Cox proportional hazards model and Kaplan–Meier curve to estimate progression-free survival (PFS) and overall survival (OS) following NIC treatment and surgery.

**Results:**

Overall, 225 patients with LAESCC were divided into training (157) and test set (68) (7:3). After a median follow-up of 2.86 years, death was observed as a positive event in 41 patients (26.1%). It is statistically significant to construct a prediction model combining radiomics features pre- and post-NIC with clinical features to predict the PFS and OS of LAESCC. The combined model showed the highest performance in predicting both disease-free survival and OS compared with the clinical or radiomics models. multivariate Cox regression analysis identified smoking (HR = 1.417, 95% confidence interval [CI]: 0.875–2.293, p = 0.156), Ki67(HR = 2.426, 95% confidence interval [CI]: 1.506–3.908, p = 0.000) and postRad-S1 (HR = 1.867, 95% CI: 1.053–3.311, p = 0.033) as significant independent covariates associated with high PFS. While Ki67 and postRad-S2 were prognostic factors significantly associated with OS (HR = 1.521, 95% CI: 0.821–2.818, p = 0.183; HR = 1.912, 95% CI: 1.001–3.654, p = 0.050, respectively).

**Conclusion:**

For patients with LAESCC treated with NIC followed by surgery, the combined model effectively evaluated the efficacy of NIC and predicted PFS and OS. Additionally, different independent predictors were associated with PFS and OS, providing clues for future studies.

## 1 Introduction

Approximately 90% of oesophageal squamous cell carcinoma (ESCC) cases occur in East Asia, nearly half (50%) of these cases concentrated in China, particularly in the Henan, Hebei, and Jiangsu provinces. ESCC is often insidious, with approximately 50% of patients diagnosed at a locally advanced stage (1). For clinical stage T4 ESCC definitive chemoradiotherapy is the primary approach comprising of high-dose radiation and chemotherapy as curative treatment. Neoadjuvant chemoradiotherapy (nCRT) or neoadjuvant chemotherapy(nCT), followed by radical surgery, has emerged as an alternative treatment for LAESCC. With the widespread implementation of neoadjuvant therapy for esophageal squamous cell carcinoma,the 5-year survival rate for advanced esophageal cancer has increased to 47% (2).

However, there is a lack of sufficient studies demonstrating that nCRT offers a significant advantage over nCT in terms of overall prognosis for ESCC (3). While neoadjuvant therapy has improved the overall survival rates among patients with localized esophageal cancer, both neoadjuvant chemotherapy and chemoradiotherapy have been associated with increased surgical challenges and poorer prognoses in many patients who do not achieve pathological complete responses (PCR) (4).

The tumor microenvironment(TME) of ESCC is primarily composed of immune cells, fibroblasts, tumor cells, and stromal components (5). This microenvironment can induce immunosuppression, thereby inhibiting the immune system’s ability to eliminate tumor cells (6). Neoadjuvant therapy has been shown to remodel the TME effectively. Specifically, radiotherapy can upregulate programmed death ligand 1(PD-L1) expression on myeloid cells, which contributes to radioresistance (7). Currently, chemotherapy can alter signaling pathways in B cells and T cells, leading to drug resistance (7). Recent studies have demonstrated that blocking the programmed death-1 (PD-1) receptor pathway has yielded promising outcomes in various cancers (8). PD-1 is expressed on different immune cells, while PD-L1 is present on tumor cells and antigen-presenting cells (9). The binding of PD-L1 to PD-1 results in T cell exhaustion and dysfunction. Camrelizumab, an antibody targeting PD-L1, enhances the body’s immune response against tumor cells by inhibiting PD-L1 function (10). It has emerged as a first-line immunotherapy for several cancers, including ESCC.Therefore, neoadjuvant immunochemotherapy (NIC) combined with radical surgery has emerged as the first-line treatment for advanced ESCC in China, Japan, and South Korea (11, 12).

However, there are only a few small-sample studies on the efficacy evaluation and long-term survival prediction of NIC combined with radical surgery for LAESCC.This highlights the need for further research (13–15). Determining the course of NIC has also been controversial (16). Currently, several studies have indicated that albumin-bound paclitaxel is associated with a reduced incidence of adverse reactions when compared to other chemotherapy agents. Furthermore, the rates of R0 resection have shown significant improvement. The combination of camrelizumab with albumin-bound paclitaxel and cisplatin may enhance long-term survival in patient[ (2, 17).In this retrospective study, we evaluated 225 patients with cT3–cT4a ESCC, who received one to four cycles of camrelizumab plus albumin-bound paclitaxel/cisplatin followed by radical surgery. We analysed pre- and post-treatment contrast-enhanced computed tomography (CT) images, pathological parameters, and long-term follow-up to investigate the relationships between clinical parameters, treatment duration, response, and survival. A comprehensive model was developed to predict patient survival by integrating clinical features, immunohistochemical characteristics, and radiomics features, which significantly improves the ability to make informed clinical decisions.

## 2 Materials and methods

### 2.1 Patient selection

**Figure 1:**
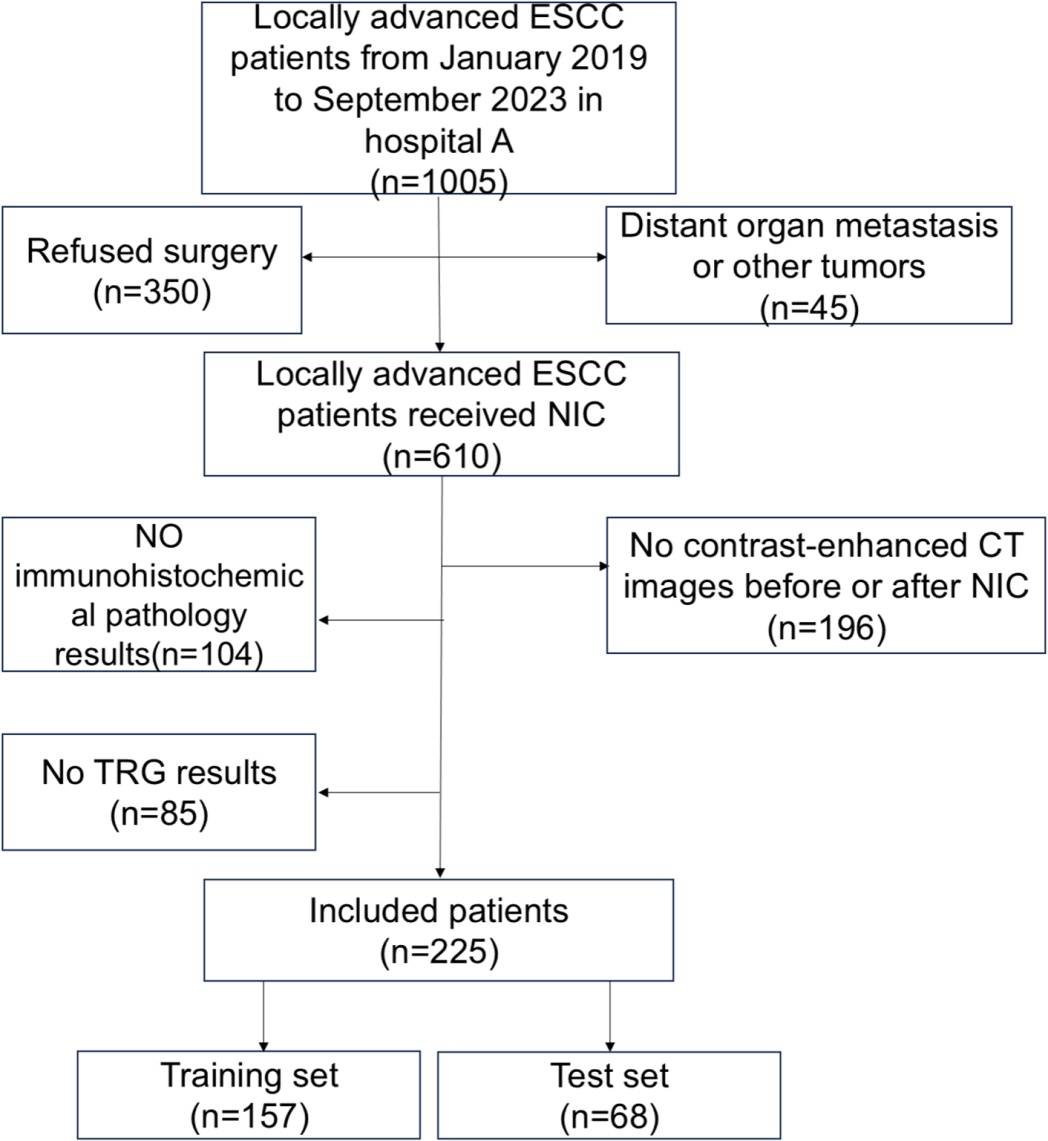
Flowchart of enrolled patients. There were 1005 patients who met the inclusion and exclusion criteria. A total of 610 patients received neoadjuvant immunochemotherapy(NIC), whereas 225 patients received enhanced CT scans before and after NIC.

This retrospective analysis included 225 consecutive patients treated at the affiliated Huai’ an No. 1 Hospital of Nanjing Medical University. All patients with cT3–cT4a ESCC without distant organ metastasis and underwent NIC followed by radical surgery from January 2019 to September 2023 were included. Patients with histologically confirmed stage 3-4a ESCC were reviewed by an experienced Thoracic Surgeon and a Radiologist(**Figure 1**). Our study received approval from the institutional review board(approval number: KY-2022-045-01).

The patient selection criteria were as follows: (1) ESCC confirmed by gastroscopy pre-treatment, and cT3–cT4a confirmed by enhanced CT. (2) No distant organ metastasis or other tumours. (3) Patients that underwent enhanced CT examinations of the neck, chest, and upper abdomen before and after NIC treatment. (4) The patients adopted NIC regimens. The exclusion criteria were as follows: (1) Incomplete CT data. (2) Incomplete immunohistochemical data.(3) Incomplete survival information. (4) Patients with other tumours.

### 2.2 Image acquisition and tumour segmentation

**Figure 2:**
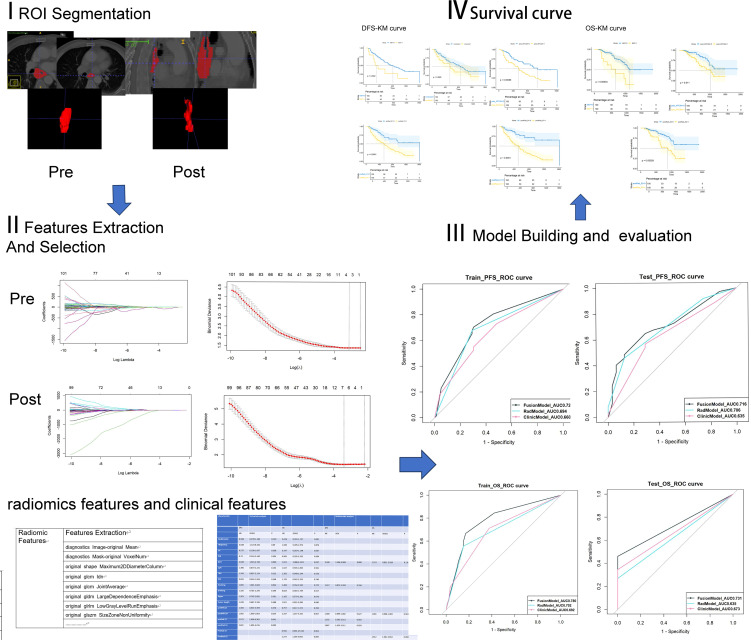
The process of radiomics analysis involves the development of clinical models, radiomics models, and fusion models to predict survival outcomes, as well as the generation of Kaplan-Meier curves.

All patients underwent contrast-enhanced CT from the lower neck to the upper abdomen before each course of NIC. We selected two CT-enhanced images of the same patient: one before NIC and another before surgery (pre- and post-NIC images respectively). The region of interest (ROI) of the lesion was semi-manually delineated using ITKsnap (version 4.0.1; http://www.itksnap.org/pmwiki/pmwiki.php) by Reader1 and Reader2. The ROI contained all tumours, avoiding gas in the lesion. Initially, an outline of the tumour was drawn using enhanced CT images before NIC. The pre-NIC target area was used as a reference for the post-NIC target area to ensure the same target area. Thus, the target area before and after the treatment remained unchanged. Mapped lesions were reviewed by a radiologist. The radiomics flowchart is described in **Figure 2**.

The CT scanning equipment used was a Siemens Dual-Source 2nd generation SOMATOM Definition Flash CT. All patients underwent enhanced CT scans of the neck, chest, and upper abdomen in the supine position, with their arms above their heads. After injecting 1.5 mL/kg contrast medium (loversol) using a high-pressure syringe at a rate of 3.0 mL/s, the scan was delayed for 30 s. The scan parameters were as follows: (1)The tube voltage of 120 kVp, (2) Automated tube current modulation, (3)Rotation time of 0.5 s, (4)Detector collimation set at 64 × 1.2 mm, (5)Slice thickness of 5 mm, (6) Matrix was 512 × 512.

The voxel sizes of contrast-enhanced CT images were not resampled. The pre- and post-NIC lesions described by Reader1 and Reader2 were detected using intra-group correlation coefficient(ICC), respectively. ICC scores greater than 0.75 pre- and post-NIC indicated good consistency. Using the ‘Jupyter’ subprogram in ‘Anaconda Navigator’ software, we extracted radiomic features of the ROI using the ‘pyradiomics’ package in Python (Version: 3.11.5). Each case yielded 130 features, and radiomic filters were applied to the original and masked images. Finally, a 225 x130 feature matrix was obtained. R software (version 4.3.2) was used for data processing. To standardise the data and remove dimensional discrepancies, min-max normalisation was employed to pre-process the data, ensuring that it was uniformly distributed within the range of [0,1].

Min-max normalisation formula:


$$xnormalized=\frac{x-\min\left(x\right)}{\max\left(x\right)-\min\left(x\right)}$$

**Figure 3:**
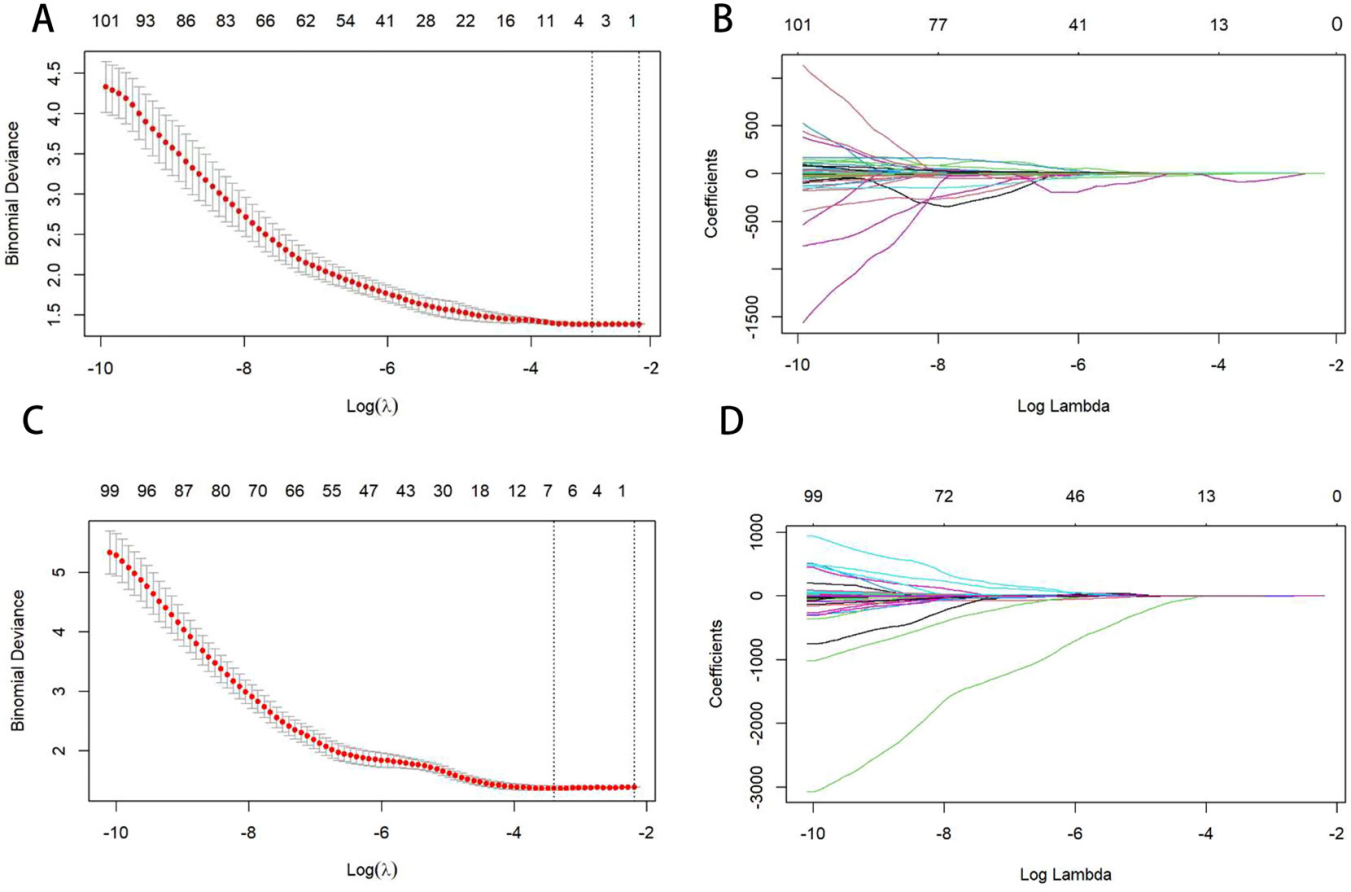
Figures **(A, C)** show the selection of the optimal LASSO-Cox model parameters using 10-fold cross-validation. The vertical axis is the partial likelihood bias (λ), which quantifies the cross-validation bias. The horizontal axis represents the log (λ) value. The upper end indicates the number of corresponding features. The vertical line on the left indicates the parameter values corresponding to the minimum deviation in cross-validation, and the vertical line on the right indicates the parameter values corresponding to a more concise model within the standard error. In this study, the optimal parameter corresponding to the first vertical line on the left is taken, that is, the error is minimal. Figures **(B, D)** are curves of the feature coefficients of radiomics features under the parameters corresponding to Figures **(A, C)**. The vertical line corresponds to the vertical line on the left of Figures **(A, C)**, indicating the optimal parameter values. Figures **(A, B)** represent pre-NIC; Figures **(C, D)** represent post-NIC.

The independent samples t-test was used to identify potentially significant features, excluding radiomic features that showed no statistical difference (p < 0.05). Finally, the least absolute shrinkage and selection operator (LASSO) method was employed to further reduce the dimensions of the features. The weight of each feature on the survival outcome of ESCC was considered, resulting in compression of the unimportant variable weights to zero. Following a 10-fold cross-validation, two λ values were selected to identify the core variables with greater weight. Moreover, the optimal features ( $x_{i}$ ) and weight coefficients ( $\beta_{i}$ ) were incorporated into the radiomics score formula to compute the Radiomics score(Rad-S) for each individual (**Figure 3**).

Radiomics score formula:


$$Radiomics\;score=\beta_{0}+\beta_{1}\;x_{1}+\beta_{2}x_{2}+\cdots \beta_{i}x_{i}$$

### 2.3 Pathological data and survival information collection

The following information was extracted from electronic medical records: age, sex, and immunohistochemical pathological results. Pathological diagnosis was determined according to Japanese Oesophageal Cancer (11^th^ edition) guidelines (18). A pathologist with extensive experience made the diagnosis. The following classification was used: keratin was determined based on the pathological results, with positive results recorded as 1 and negative results recorded as 0. Lymphatic vessel invasion (Lvi) was determined based on immunohistochemical results, with positive results recorded as 1 and negative results recorded as 0. Ki67 overexpression (>70%) was recorded as 1, while Ki67 non-expression and low expression (≤70%) were recorded as 0. P53 overexpression (≥40%) was defined as 1, and P53 negative or low expression (0–39%) was defined as 0 (19).

The primary endpoints were progression-free survival (PFS) and the secondary endpoint was overall survival (OS). Survival, recurrence, and metastasis was monitored via telephone contacted with patients every three months. Patients who survived >3 years underwent annual physical examinations at our hospital’s outpatient department. PFS was defined as the time from the initial surgery to the end of the surgery, while OS was defined as the time from primary surgery to death from all causes.

### 2.4 NIC regimen and esophagectomy

All patients received one–four cycles of immunochemotherapy. Albumin-bound paclitaxel was administered at a dose of 175 mg/m^2^, and cisplatin was administered at 75 mg/m^2^. Simultaneously, 200 mg of camrelizumab was administered intravenously. Each course of medication was administered over 1–4 days, and minimally invasive thoracoscopic radical surgery was performed 3–4 weeks after the completion of the last cycle.

### 2.5 Statistics

Radiomic feature extraction was performed using the ‘pyradiomics’ package in PyCharm (Python version 3.13.1). Statistical analyses were conducted using R software (version 3.6.3), utilizing the following packages: “caret”, “survival”, “rms”, and “ggplot2”.

Continuous variables were described using mean ± standard deviation, while categorical variables were analysed using frequencies and percentages. Comparisons between groups were conducted using independent-sample t-tests or Mann-Whitney U tests for continuous variables and the chi-square test for binary or categorical variables. OS was defined as the time from the day of surgery to the date of death. PFS was defined as the duration between the day of surgery and the first detection of local recurrence, metastasis, or death, regardless of the cause.

Univariate Cox regression models were used to examine the relationship between survival and clinical variables, including pre-immunochemotherapy, post-immunochemotherapy, and postoperative pathological variables. After testing in a univariate model, a multivariate Cox regression model was generated using variables with P<0.05 to estimate the patient survival associated with the variables.A p-value less than 0.05 was statistically significant.

Kaplan–Meier curves were used to determine the PFS and OS. Log-rank analysis was used to assess differences in survival between the various patient groups.

## 3 Results

### 3.1 Clinical baseline features

A total of 225 patients with LAESCC received NIC, followed by radical surgery. The patients were randomly divided into training and test sets in a 7:3 ratio. There were no significant differences in the statistical comparison of basic clinical features between the two sets (**Table 1**). We utilized Rad-S before and after the NIC to predict DFS and OS. Meanwhile, PreRad-S1 and PostRad-S1 were applied in PFS models, while PreRad-S2 and PostRad-S2 were utilized in OS models.

**Table 1:** Clinical demographic data in the training and test cohorts.

Characteristics	Training data (157)	Test data (68)	P
Age (year)	66.3 ± 6.376	65.31 ± 6.273	0.882
	67 (62-70)	66 (62-70)	
Gender			
male	114 (72.6%)	52 (76.5%)	
female	43 (27.4%)	16 (23.5%)	0.622
Tumor length (mm)	63.33 ± 20.45	61.49 ± 20.15	0.688
	60 (50-72)	60 (49-75)	
Pre-MTCSA (mm2)	470.71 ± 257.27	426.85 ± 151.79	0.214
	440.44 (337.83-548.85)	432.86 (316.61-515.90)	
Post- MTCSA (mm2)	251.88 ± 148.45	231.47 ± 105.21	0.118
	222.77 (155.94-288.68)	202.86 (168.67-271.76)	
ypN			0.379
Yes (0)	94 (59.9%)	36 (32.9%)	
No (≥1)	63 (40.1%)	32 (47.1%)	
pCR			0.521
Yes	22 (14%)	7 (10%)	
No	135 (86%)	61 (90%)	
Lvi			0.128
Yes (0)	141 (89.8%)	56 (82.4%)	
No (1)	16 (10.2%)	12 (17.6%)	
PNI			0.804
Yes (0)	143 (91.1%)	61 (89.7%)	
No1 (1)	14 (8.9%)	7 (10.3%)	
Ki67			0.879
Yes (≤70%)	101 (64.3%)	45 (66.2%)	
No (>70%)	56 (35.7%)	23 (33.8%)	
P53			0.772
Yes (0)	87 (55.4%)	36 (52.9%)	
No (1)	70 (44.6%)	32 (47.1%)	
Cycles of chemotherapy			0.335
1	30 (19.1%)	8 (11.8%)	
2	118 (75.2)	51 (75%)	
3	9 (5.7%)	8 (11.7%)	
4	0	1 (1.5%)	
Cycles of immunotherapy			0.112
1	132 (84.1%)	58 (85.3%)	
2	22 (14%)	9 (13.2%)	
3	3 (1.9%)	1 (1.5%)	

MTCSA, Mean tumor cross sectional area; LVI, lymphatic vessel invasion; pCR, Pathological Complete Response; Ki67, expression level; P53, mutation status; ypN, lymph node after neoadjuvant therapy; PNI, perineural invasion.

**Figure 4:**
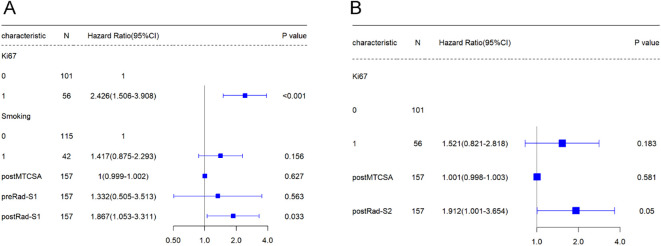
The forest plot represents the results of the Multivariable Cox regression analysis conducted in the training cohort.

In **Table 2**, univariate Cox regression revealed that Ki67, smoking, post-mean tumour cross-sectional area (post-MTCSA), preRad-S1, and postRad-S1 were correlated with PFS, while Ki67, post-MTCSA, and PostRad-S2 were correlated with OS in the training set. Variables with a p < 0.05 were included in the multivariate Cox regression analysis, and their significance was as follows: Ki67 and postRad-S1 in predicting PFS, while only PostRad-S2 in predicting OS (**Figure 4**).

**Table 2:** Univariable and multivariable Cox regression analysis in the traing cohort according to clinical features and radiomics scores.

Characteristic		Univariate analysis						Multivariate analysis				
	PFS			OS			PFS			OS		
	HR	95%CI	P	HR	95%CI	P	HR	95%	P	HR	95%CI	P
Sex(female)	0.568	0.278-1.160	0.120	0.476	0.201-1.127	0.091						
TRG	3.048	1.124-8.262	0.09	1.083	0.395-2.973	0.876						
Lvi	0.575	0.198-1.667	0.308	0.147	0.019-1.148	0.067						
PNI	0.74	0.244-2.240	0.594	0.995	0.295-3.353	0.994						
Ki67	3.028	1.924-4.766	0.000	1.811	1.008-3.2511	0.047	2.426	1.506-3.908	0.000	1.521	0.821-2.818	0.1 83
ypN	1.396	0.873-2.231	0.164	1.027	0.565-1.865	0.931						
pCR	1.343	0.837-2.154	0.222	1.205	0.648-2.244	0.556						
P53	0.832	0.443-1.563	0.568	1.125	0.562-2.253	0.740						
Smoking	1.665	1.041-2.663	0.033	1.406	0.762-2.592	0.275	1.417	0.875-2.293	0.156			
Drinking	1.326	0.781-2.250	0.296	0.823	0.383-1.769	0.617						
Hypertesion	1.263	0.765-2.082	0.361	1.165	0.570-2.381	0.676						
Tumor length	1.228	0.687-2.196	0.488	1.921	0.925-3.990	0.080						
preMTCSA	1.458	0.633-3.359	0.376	0.757	0.417-1.372	0.359						
postMTCSA	1.002	1.000-1.003	0.034	2.337	1.257-4.345	0.007	1.000	0.999-1.002	0.627	1.001	0.998-1.003	0.581
preRad-S1	2.573	1.038-6.381	0.041				1.332	0.505-3.513	0.563			
postRad-S1	2.601	1.605-4.216	0.000				1.867	1.053-3.311	0.033			
PreRad-S2				6.550	0.901-47.610	0.063						
PostRad-S2				2.273	1.263-4.092	0.006				1.912	1.001-3.654	0.050

PFS, progression-free survival; OS, overall survival; 95%CI, 95% confidence interval; HR, hazard ratio; TRG, tumor trgression grade; LVI, lymphatic vessel invasion; Ki67, expression level; P53, mutation status; ypN, lymph node after neoadjuvant therapy; pCR, Pathological Complete Response; PNI, perineural invasion; Rad-S, radiomics-scores. p ≤ 0.05 is considered statistically significant.

### 3.2 Radiomics features and Rad scores

Three wavelet CT image filters were used in the feature extractor. A total of 123 non-wavelet CT image-omics features were obtained, including 15 diagnostic, 14 shape, 19 first-order statistical, 24 grey co-occurrence matrix, 14 grey dependence matrix, 16 grey run matrix, 16 grey size area matrix, and five neighbourhood grey tone difference matrix features. Two pre-NIC and six post-NIC features for predicting PFS were selected using the t-test and the Least Absolute Shrinkage and Selection Operator(LASSO). Two pre-NIC features and six post-NIC features for predicting OS were selected using a t-test and the LASSO (**Supplementary 1**). The Rad scores utilised in the PFS and OS prediction models were calculated using pre- and post-NIC screened features, and the coefficients were then derived. We designated the Rad-score pre- and post-NIC in the PFS model as preRad-S1 and postRad-S1, respectively, and as preRad-S2 and postRad-S2 in the OS model.

### 3.3 Clinical features associated with survival

At the end of the study period, 154 patients were still alive, 69 had succumbed to ESCC-related issues, while two had died of other causes. The median postoperative survival time was 651 days (range = 177–1676 days). We used the Cox proportional hazard ratio (HR) to extract significant independent prognostic factors from the clinical and pathological features of OS and PFS.

Univariate Cox regression analysis showed that smoking, Ki67, post-MTCSA, preRad-S1, and postRad-S1 were prognostic factors significantly associated with PFS. Additionally, Ki67, post-MTCSA, and postRad-S2 were significantly associated with OS. Subsequently, multivariate Cox regression analysis identified smoking (HR = 1.417, 95% confidence interval [CI]: 0.875–2.293, p = 0.156), Ki67(HR = 2.426, 95% confidence interval [CI]: 1.506–3.908, p = 0.000) and postRad-S1 (HR = 1.867, 95% CI: 1.053–3.311, p = 0.033) as significant independent covariates associated with high PFS. While Ki67 and postRad-S2 were prognostic factors significantly associated with OS (HR = 1.521, 95% CI: 0.821–2.818, p = 0.183; HR = 1.912, 95% CI: 1.001–3.654, p = 0.050, respectively).

### 3.4 Constructing a model to predict PFS and OS

Smoking, Ki67, post-MTCSA, preRad-S1, and postRad-S1 were used to construct a combined model to predict PFS, whereas Ki67, post-MTCSA, and postRad-S2 levels were used to construct a combined model to predict OS (**Table 3**). As for the prediction of PFS, the C-index of the fusion model (smoking, Ki67, and postRad-S2) was 0.725 (95% CI: 0.648–0.764), which was higher than the C-index of the clinical model (smoking and Ki67) (C-index = 0.645,95% CI: 0.597–0.723) and the Rad model (C-index = 0.648, 95% CI: 0.597–0.699) in the training set. In the test set, the C-indices for the Fusion, clinical, and Rad models were 0.620, 0.595, and 0.592, respectively. We calculated the variable importance score for each variable by dividing the absolute value of its coefficient by the total sum of absolute regression coefficients.

**Table 3:** Prediction models for OS and PFS based on covariates in the traing cohort, including the error bounds, 95% confidence intervals, hazard ratios, and P-values.

Model	Covariate	Variable Importance Score	Coefficient	HR (CI)	P value
PFS					
Rad-model	preRad-S1	0.217	0.489	0.612 (0.282-1.329)	0.215
	postRad-S1	0.782	1.756	5.791 (2.445-13.712)	<0.001
Clinic-model	Smoking	0.216	0.485	1.623 (1.012-2.602)	0.044
	Ki67	0.421	0.947	2.578 (1.226-5.418)	0.012
	post-MTCSA	0.363	0.815	2.258 (1.421-3.590)	<0.001
Fusion-model	preRad-S1	0.177	0.763	0.466 (0.209-1.039)	0.062
	postRad-S1	0.420	1.805	6.077 (2.573-14.354)	<0.001
	Smoking	0.123	0.531	1.699 (1.056-2.734)	0.029
	Ki67	0.190	0.817	2.264 (1.061-4.829)	0.034
	post-MTCSA	0.089	0.386	1.471 (0.861-2.512)	0.157
OS					
Rad-model	postRad-S2	1	1.049	2.853 (1.581-5.150)	<0.001
Clinic-model	Ki67	0.623	1.282	3.605 (1.728-7.517)	<0.001
	post-MTCSA	0.377	0.776	2.170 (1.195-3.951)	0.011
Fusion-model	postRad-S2	0.317	0.748	2.112 (1.123-3.972)	0.020
	Ki67	0.425	1.003	2.725 (1.267-5.862)	0.010
	post-MTCSA	0.258	0.609	1.839 (0.993-3.404)	0.052

PFS, progression-free survival; OS, overall survival; MTCSA, Mean tumor cross sectional area; Ki67, expression level; CI, confidence interval; HR, hazard ratio; Rad-S, radiomics-scores; preRad-S1 and postRad-S1 applied in PFS models; preRad-S2 and postRad-S2 applied in OS models. p ≤ 0.05 is considered statistically significant.

**Figure 5:**
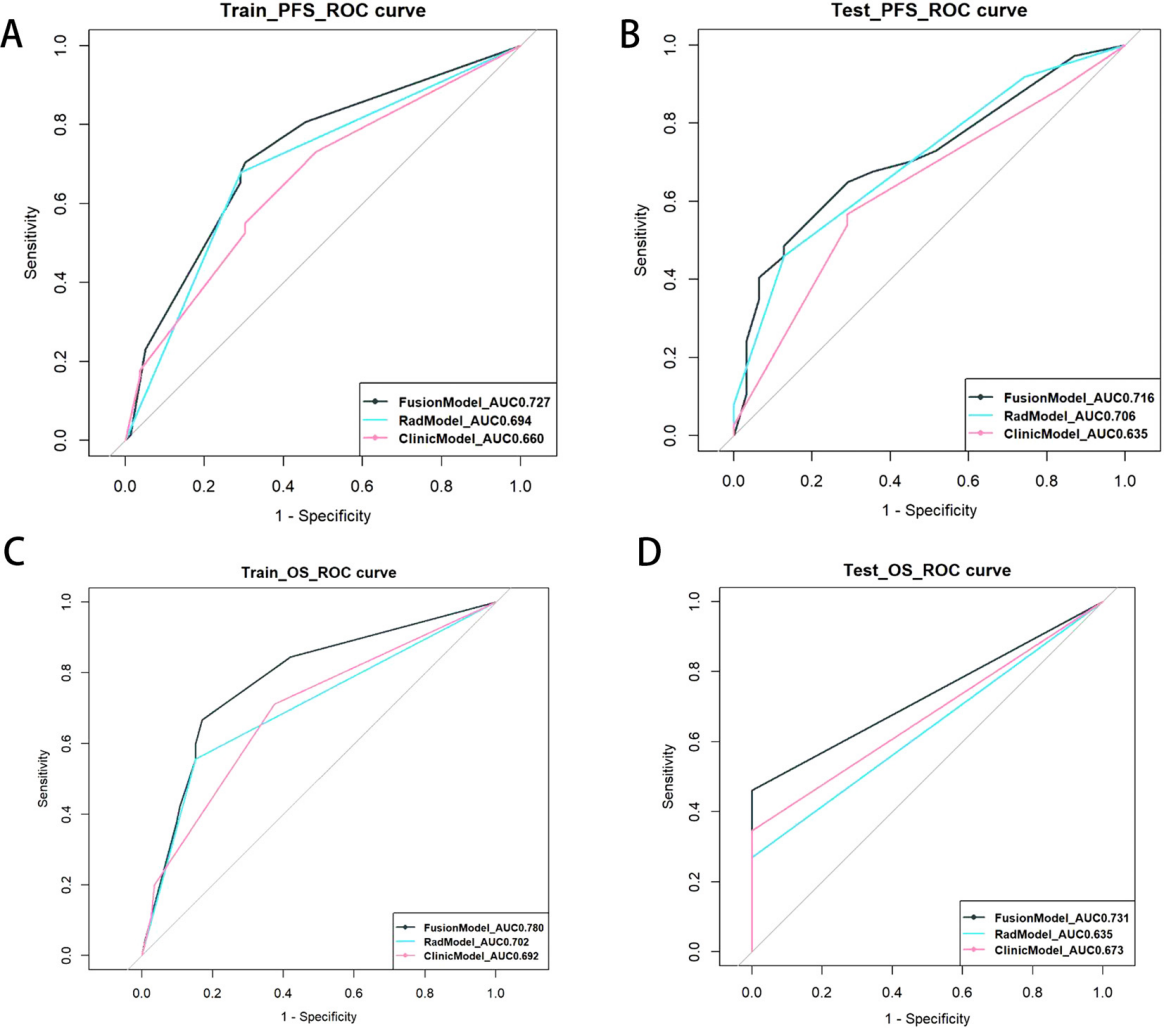
ROC curve of survival on PFS (traing set **(A)** and test set **(B)**) and OS(traing set **(C)** and test set**(D)**). Each subgraph contains clinical, radiomics, and fusion prediction model.

For the prediction of OS, the C-index of the fusion model was 0.721 (95% CI: 0.651–0.819), which outperformed the clinical models (C-index = 0.680, 95% CI: 0.617–0.775) and the Rad model (C-index = 0.626, 95% CI: 0.558–0.721) in the training set. In the test set, the C-indices of the three models were 0.670, 0.623, and 0.585, respectively (**Table 4**). To assess prognostic performance(**Figure 5**) of the PFS and OS models, we used receiver operating characteristic curves and area under the curve (AUC) values.

**Table 4:** The prediction performance of three models in the training and test sets, denoted by C-index.

PFS	C-index	
	Training set (157)	Test set (68)
Rad-model	0.648 (0.597-0.699)	0.592 (0.486-0.698)
Clinic-model	0.645 (0.597-0.723)	0.595 (0.500-0.693)
Fusion-model	0.725 (0.648-0.764)	0.620 (0.510-0.731)
OS		
Rad-model	0.626 (0.558-0.721)	0.585 (0.493-0.676)
Clinic-model	0.680 (0.617-0.775)	0.623 (0.512-0.730)
Fusion-model	0.721 (0.651-0.819)	0.670 (0.556-0.781)

PFS, progression-free survival; OS, overall survival; Rad-S, radiomics-scores.

### 3.5 Association between Ki67, smoking, post-MTCSA, preRad-S1, postRad-S1, and PFS

**Figure 6:**
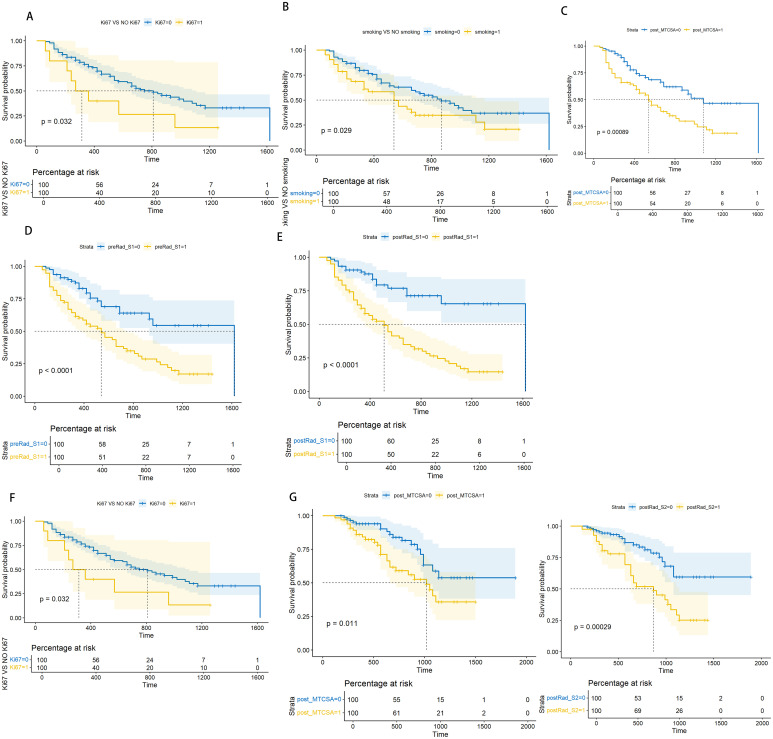
Log-rank test based on Kaplan-Meier curve. Kaplan-Meier curve for PFS prediction based on Ki67 level(Ki67), with a Log-rank test P-value of 0.039 **(A)**. Kaplan-Meier curve for PFS prediction based on smoking level, with a Log-rank test P-value of 0.029 **(B)**. Kaplan-Meier curve for PFS prediction based on post_MTCSA, with a Log-rank test P-value of 0.00089 **(C)**. Kaplan-Meier curve for PFS prediction based on preRad_S1, with a Log-rank test P-value <0.0001 **(D)**. Kaplan-Meier curve for PFS prediction based on postRad_S1, with a Log-rank test P-value <0.0001 **(E)**. Kaplan-Meier curve for OS prediction based on Ki67 level, with a Log-rank test P-value of 0.00034 **(F)**. Kaplan-Meier curve for OS prediction based on post_MTCSA, with a Log-rank test P-value of 0.011 **(G)**. Kaplan-Meier curve for OS prediction based on postRad_S2 with a Log-rank test P-value of 0.00029 **(H)**.

The Kaplan–Meier curve among Ki67, smoking, post-MTCSA, preRad-S1, and postRad-S1 with p < 0.05 was plotted after univariate and multivariate Cox regression analyses. The log-rank test showed that the PFS outcomes of each group were different, with p-values of 0.032, 0.029, 0.00089, <0.0001, and <0.0001 (**Figure 6**).

### 3.6 Association between Ki67, post-MTCSA, preRad-S2, and OS

In Cox regression analysis, both univariate and multivariate analyses revealed significant statistical differences in OS among Ki67, post-MTCSA, and preRad-S2. By constructing the Kaplan–Meier curves, the p-values obtained from the log-rank test were 0.00034, 0.011, and 0.00029, respectively (**Figure 5**).

## 4 Discussion

In this retrospective study, we developed a CT-enhanced imaging omics model incorporating clinical indicators to predict the survival of patients with locally advanced ESCC who received neoadjuvant chemotherapy combined with immunotherapy. According to the multicentre RCT trial KEYNOTE-590, immunotherapy has gradually become the first-line treatment for locally advanced ESCC. Consequently, predicting the pathological response rate and survival outcomes of preoperative neoadjuvant chemotherapy combined with immunotherapy has become a key research focus. Therefore, the identification of reliable predictive markers is crucial. Lu et al. studied patients with LAESCC who received neoadjuvant chemotherapy(NACT) and found that the OS of patients who received NACT combined with adjuvant chemotherapy was significantly higher than that of patients who received NACT alone (55.8% vs. 39.5%, p = 0.039) (20). Recent studies have demonstrated that programmed cell death ligand 1, circulating tumour DNA, serum lactate dehydrogenase, the lymph node ratio (LNR), pathologic lymph nodes staging after neoadjuvant therapy (ypN), lymphovascular invasion, and tumour length are predictors of regression grade and long-term survival after neoadjuvant therapy in advanced oesophagus cancer. Ruan et al. analysed postoperative pathological indicators but found no statistically significant difference in Lvi, ypN, or perineural invasion (PNI) (21). Sugase et al. reported a different finding: among ypN-positive patients after neoadjuvant therapy, the PFS time of patients receiving NIC was significantly longer than that of patients receiving chemotherapy (22). Chen et al. found a superior 3-year OS rate in patients with a low LNR than in those with a high LNR (81.7% vs. 55.3%; p < 0.001). Similarly, a higher PFS rate was observed in the low LNR group than in the high LNR group (79.9% vs. 37.4%; p < 0.001) (23). Yang et al. found that major pathologic response was considered an independent prognostic factor for ESCC recurrence after NIC (HR 0.39; 95% CI 0.21–0.82; p < 0.05). In univariate Cox analysis, they found that sex (p = 0.035), TNM stage (p = 0.051), tumour length after treatment (p = 0.001), and ADC value after treatment were risk factors for OS after NIC for LAESCC (24). However, multivariate Cox regression analysis showed that only the ADC value after NIC was still statistically significant (p = 0.05). However, the sample size of this study was only 30 cases (25).

However, in our study, CPR, Lvi, ypN, and PNI were excluded from the univariate analysis because they had no significant correlation with survival. The discrepancies might stem from the utilization of diverse NIC regimens among patients across various studies. Nevertheless, in our study, all patients enrolled received camrelizumab plus albumin-bound paclitaxel/cisplatin. Therefore, in the subsequent study, we aim to explore whether there are differences in survival predictors in patients with ESCC who receive different NIC treatment regimens.

Additionally, there were other clinical indicators of concern. In univariate Cox regression analysis, Ki67, smoking, and post-MTCSA were all correlated with PFS, while Ki67 and smoking were correlated with OS. In the multivariate Cox analysis, only Ki67 remained statistically significant in PFS. Ki67 and post-MTCSA showed statistical significance in OS. In previous studies, maximum tumour diameter has been proven to be an independent predictor of OS (26). In our study, we calculated the volume of the tumour and divided it by the upper and lower diameter ranges of the lesion to obtain the index of the MTCSA, which has a significance similar to the maximum tumour diameter. However, in the oesophageal cavity, the tumour shape was irregular, and the maximum tumour diameter was determined by the supervisor during measurement. The objective measurement indicators, although the measurement of MTCSA is more objective.

However, high Ki67 expression is associated with advanced tumour staging in ESCC (27). Our findings indicates that Ki67 can serve as a significant independent prognostic marker, consistent with previous studies. A recent study has revealed that bacteria residing in immunosuppressive micro-niches can promote elevated Ki-67 expression in tumour cells, impairing the immune response of the oesophageal tissue, leading to tumour proliferation. Ki67 is a well-known index marker of invasive tumour behaviour, including dedifferentiation (28). Our study demonstrated a significant association between high expression Ki67 and OS. ESCC patients with high Ki67 expression after NIC treatment had lower survival rates in terms of OS and PFS, which were statistically significant in predicting recurrence or metastasis. This indicated the substantial long-term prognostic significance of Ki67 in LAESCC.

Ki67, synthesised at the onset of cell proliferation, is expressed in all phases of the cell cycle except phase G0. It is a proliferation-associated nuclear protein and serves as a biomarker for various cancers (29). Studies of head and neck squamous cell carcinoma have demonstrated that high Ki67 expression correlates with poor OS and an increased risk of lymph node metastasis in the head and neck region (30, 31). Currently, there is no globally agreed standard definition for Ki67. It is widely accepted that high levels of Ki67 respond well to adjuvant therapy, while low levels indicate a less favorable response (32). The immunohistochemical findings of this study were derived from NIC treatment, and survival outcomes were predicted based on post-treatment Ki67 results. We experimented with various cut-off values, including 10%, 20%, 30%, 40%, 50%, 60%, 70%, 80%, and 90%, and ultimately determined that a cut-off value of 70% yielded statistically significant results in survival analysis. Using 70% as the cut-off value for predicting survival after NIC treatment, this needs to be verified by more centers.

In our study, the radiomics score, a novel quantitative index, was utilized to predict the PFS and OS.

Luo et al. analysed plain CT images of 221 patients with LAESCC before receiving concurrent chemoradiotherapy and developed a Rad-score model based on 17 radiomic features to predict local PFS. The C-index in the training cohort was 0.745 (95% CI 0.7700–0.790) (33). Li et al. used a delta model constructed with eight CT-enhanced radiomic features to predict the response rate in 95 patients with LAESCC treated with NIC, achieving an AUC of 0.848 (95% CI: 0.765–0.917) (34). Zhang et al. studied the survival of 82 patients with ESCC who received NIC treatment before surgery and built a prediction model by extracting the image-omics features of 17 pre-treatment CT-enhanced images, and the AUC obtained was 0.93 (0.87–0.99) (35). Ruan et al. investigated 192 patients with LAESCC who received preoperative NIC treatment and established a prediction model based on the radiomics score constructed by the screened four preoperative CT-enhanced image features and concluded that high RFS was correlated with negative vascular infiltration (p = 0.038), while OS was not correlated with negative vascular infiltration (p = 0.310) (21). Zhu et al. collected CT images of 64 patients with LAESCC and screened five radiomic features (Wavelet_HLL_firstorder_Skewness,Wavelet_LHL_firstorder_Maximum,Wavelet_LLH_glcm_ClusterProminence, Wavelet_LHL_gldm_DependenceVariance, and Original_glszm_SizeZoneNonUniformity) to build a model to predict the efficacy of NIC treatment before surgery, achieving a significant effect (p = 0.0059) (36).

In previous studies, MR radiomics models have been used to predict survival in ESCC. Chu et al. selected three image-omics features from MRI-contrast-enhanced imaging of 434 patients with ESCC to develop a radiomics-combined clinical model for predicting PFS (C-index = 0.714, 95% CI = 0.673–0.753) and five features combined with a clinical model for predicting OS (C-index = 0.730, 95% CI, 0.687–0.773). Using the radiomic model alone, the C-indices for PFS and OS were significantly lower at 0.641 (95% CI, 0.602–0.680) and 0.649 (95% CI, 0.602–0.697), respectively (37). Jin et al. observed similar outcomes in their investigation of the response of patients with ESCC to nCRT, with the combined clinical model of radiomics demonstrating a significantly higher C-index than the radiomics alone model. These findings are consistent with those of our study, indicating that the predictive power of the radiomic model alone was inferior to that of the combined radiomic and clinical models (38).

The results of both CT-enhanced and MR radiomics demonstrated that the combined model had outperformed the single radiomics and the single clinical models in predicting survival. In comparing the CT-enhanced images before and after NIC treatment, imaging omics scores were found to be statistically significant in predicting PFS at both time points. However, only the post-treatment imaging omics scores exhibited statistical significance in predicting OS. This indicates that post-NIC imaging data can reflect both PFS and OS, whereas pre-NIC imaging data can only reflect PFS. Incorporating clinical information may further improve the predictive accuracy of imaging omics scores.

The radiomic features selected by pre-NIC in our study included shape features (minimum axis length), grey co-occurrence matrix, and NGTDM. The latter two belong to texture features, which represent the grey level of the image and the spatial distribution characteristics of pixels and indirectly reflect the heterogeneity of the tumour. The image-omics features screened by post-NIC included two shape features (the maximum two-dimensional diameter of the object in the image and the ratio of surface area to volume), two first-order features (the ratio of surface area to volume of the object and the maximum grey value of the object), and two texture features. This suggests that imaging after treatment provides more information about tumour changes, and that post-NIC can predict both PFS and OS.

Compared with clinical features and traditional imaging features, radiomics is a unique branch of image recognition in the medical field. Expanding samples or model training combined with PET/CT,MR and pathological images can definitely predict the prognosis of ESCC patients received NIC.

Our study has some limitations. First, to increase the stability of model prediction, we only studied the survival of patients treated with neoadjuvant chemotherapy combined with immunotherapy. Owing to individual patient differences, we aim to increase the survival of patients receiving neoadjuvant radiotherapy or neoadjuvant chemoradiotherapy combined with immunotherapy in future studies, and provide more treatment options for the clinic. Additionally, this was a single-centre study, and more centres in future studies, will jointly study the survival of patients with LAESCC after neoadjuvant chemotherapy combined with immunotherapy to increase the accuracy of the model. Besides, there is still no global consensus on the biological interpretation of the radiomic model. And the differences in images produced by different devices and scanning protocols that affect the stability of the model remain unresolved.

## 5 Conclusions

Our findings demonstrate that model combining CT-based Rad and clinical factors, may improve the prediction of OS and PFS in patients with LAESCC undergoing NIC. In addition, Ki67 (cut-off: 70%) could be an important independent prognostic indicator in ESCC treated with NIC followed surgery. These could guide some patients to receive further postoperative adjuvant therapy to improve the prognosis.

## Data Availability

The original contributions presented in the study are included in the article/**Supplementary Material**. Further inquiries can be directed to the corresponding authors.
