# Supplementary material for: Prognostic factors in locally advanced oesophageal squamous cell carcinoma: a clinical and radiomic analysis of neoadjuvant immunochemotherapy before surgery
Source: Front Oncol. 2025 Mar 13;15:1508477. doi: 10.3389/fonc.2025.1508477 (PMC11966397; doi:10.3389/fonc.2025.1508477)
Supplement: Supplementary file 1 [file Table1.docx]

Supplementary 1 LASSO regression was used to finally screen the radiomics features

| Lasso and T-test features | Pre_NIC | Post_NIC |
| --- | --- | --- |
| PFS | original_shape_LeastAxisLength  (representing the minimum axial length in the original lesion) | original_shape_Maximum2DdiameterColumn  (representing the local aggressiveness, growth pattern, or prognosis of the tumor) |
|  | original_glcm_InverseVariance  (representing the texture of the lesion) | original_shape_SurfaceVolumeRatio  (representing the growth pattern of the tumor) |
|  |  | original_firstorder_Maximum  (representing the pathological features of the lesion structure) |
|  |  | original_firstorder_Median  (representing the the lesion tissue type) |
|  |  | original_glcm_Imc2  (representing the lesion structure, cell arrangement, texture pattern of the lesion, etc) |
|  |  | original_glcm_InverseVariance  (representing the texture of the lesion) |
| OS | original_shape_SurfaceVolumeRatio  (representing the complexity of the tumor or assesses the progression of the disease) | original_shape_Maximum2DDiameterSlice  (representing the type, stage, grade, or other biological characteristic of disease) |
|  | original_glcm_InverseVariance  (representing the texture of the lesion) | original_shape_MinorAxisLength  (representing the morphological changes of cells and the structural characteristics of tissues) |
|  |  | original_firstorder_Maximum  (representing the pathological features of the lesion structure) |
|  |  | original_glcm_Imc2  (representing the lesion structure, cell arrangement, texture pattern of the lesion, etc) |
|  |  | original_glcm_InverseVariance  (representing the texture of the lesion) |
|  |  | original_glcm_MCC  (representing the structure and composition of an organization) |

LASSO the Least Absolute Shrinkage and Selection Operator,NIC Neoadjuvant immunochemotherapy,

PFS progression-free survival,OS overall survival
